# Transcriptome Analysis of Neonatal Larvae after Hyperthermia-Induced Seizures in the Contractile Silkworm, *Bombyx mori*

**DOI:** 10.1371/journal.pone.0113214

**Published:** 2014-11-25

**Authors:** Hongyi Nie, Chun Liu, Yinxia Zhang, Mengting Zhou, Xiaofeng Huang, Li Peng, Qingyou Xia

**Affiliations:** 1 State Key Laboratory of Silkworm Genome Biology, Chongqing, China; 2 the Key Sericultural Laboratory of the Ministry of Agriculture, Southwest University, Chongqing, China; University of Lleida, Spain

## Abstract

The ability to respond quickly and efficiently to transient extreme environmental conditions is an important property of all biota. However, the physiological basis of thermotolerance in different species is still unclear. Here, we found that the *cot* mutant showed a seizure phenotype including contraction of the body, rolling, vomiting gut juice and a momentary cessation of movement, and the heartbeat rhythm of the dorsal vessel significantly increases after hyperthermia. To comprehensively understand this process at the molecular level, the transcriptomic profile of *cot* mutant, which is a behavior mutant that exhibits a seizure phenotype, was investigated after hyperthermia (42°C) that was induced for 5 min. By digital gene expression profiling, we determined the gene expression profile of three strains (*cot*/*cot ok*/*ok*, +/+ *ok*/*ok* and +/+ +/+) under hyperthermia (42°C) and normal (25°C) conditions. A Venn diagram showed that the most common differentially expressed genes (DEGs, FDR<0.01 and log2 Ratio≥1) were up-regulated and annotated with the heat shock proteins (HSPs) in 3 strains after treatment with hyperthermia, suggesting that HSPs rapidly increased in response to high temperature; 110 unique DEGs, could be identified in the *cot* mutant after inducing hyperthermia when compared to the control strains. Of these 110 unique DEGs, 98.18% (108 genes) were up-regulated and 1.82% (two genes) were down-regulated in the *cot* mutant. KEGG pathways analysis of these unique DEGs suggested that the top three KEGG pathways were “Biotin metabolism,” “Fatty acid biosynthesis” and “Purine metabolism,” implying that diverse metabolic processes are active in *cot* mutant induced-hyperthermia. Unique DEGs of interest were mainly involved in the ubiquitin system, nicotinic acetylcholine receptor genes, cardiac excitation–contraction coupling or the Notch signaling pathway. Insights into hyperthermia-induced alterations in gene expression and related pathways could yield hints for understanding the relationship between behaviors and environmental stimuli (hyperthermia) in insects.

## Introduction

Insects make up a substantial proportion of the global biota. Approximately 920,000 insect species have been described, which represent almost 85% of all known animal species [1]. This implies that insect have good adaptability because they can adjust their behaviors and adapt to the total environment. Generally, insect behavior encompasses three aspects: behavior with members of its own species, behavior with members of other species and behavior with the physical environment. Here, we focus on relationships between an insect and its environment.

Most insects face mal-conditions, in which temperatures are outside their physiological limits. Different species use different strategies to cope with unfavorable circumstances. Temperate-zone insects (such as *Halictus scabiosae* and *Anopheles gambiae*) can tolerate markedly different external conditions in the summer and winter, which they accomplish by winter hibernation or summer aestivation [2], [3]. The Asian tiger mosquito, *Aedes albopictus*, can respond to harsh environmental changes to survive by quiescence or diapause [4]. The former is an alternative form of insect dormancy, in which physiological processes immediately halt in response to the reduction of an environmental, physiologically limiting factor; the latter is an alternative developmental program that is initiated in response to severely hostile conditions that can last for several months [5].

As mentioned above, thermal resistance represents as important physiological trait that affects the adaptation of insects to their environments. Thermal stresses have been considered in various insect, such as locusts, whiteflies, beetles, moths, ants, fruit flies and parasitic wasps [6]. It has been suggested that constitutively expressed heat shock proteins (HSPs) might be responsible for survival in potentially lethal temperatures [7]. Alternatively, alterations in cell membrane composition or changes in allozymes or enzyme concentrations might also be involved [8]. However, the physiological basis for this variation in promoting thermotolerance is much less clear.

In previous studies, mutants have played a vital role in our understanding of physiological and behavioral changes [9]–[12]. The silkworm is a Lepidoptera model insect and has about 400 mutants; it is a precious bioresource for genetic studies.

Contractile (*cot*), which is a natural recessive behavioral mutant silkworm [13], exhibits strong contraction, rolling, vomiting and a momentary cessation of movement after manual stimulation, which is followed by a slow recovery period. In this study, we found that *cot* is a hyperthermia-induced mutant. Thus, *cot* is a well-suited model system for studying responses to hyperthermia. To comprehensively understand the interactions between the silkworm and hyperthermia, Digital Gene Expression (DGE) was performed to identify gene expression changes after hyperthermia-induced seizures in the *cot* mutant. To identify differentially expressed genes (DEGs) associated with hyperthermia in the *cot* mutant, we removed the DEGs that we also identified in control strains, *ok* (+/+ *ok*/*ok*) and Dazao (+/+ +/+). Our results showed that unique DEGs of interest in the *cot* mutant that were induced after hyperthermia were involved in the ubiquitin system, nicotinic acetylcholine receptor genes, cardiac excitation–contraction coupling and the Notch signaling pathway. These data provided new insights into the transcriptional regulation in *cot* mutant after hyperthermia-induced seizures, which offer a valuable resource for studying the mutual relationship between behavior and hyperthermia in an insect.

## Materials and Methods

### Silkworm strains

The *cot* mutant (*cot*/*cot*), *ok* (*ok*/*ok*) and Dazao (wild-type, +/+) strains were kindly provided by the Silkworm Gene Bank of Southwest University (Chongqing, China). To obtain the transparent cuticle *cot* mutant, a cross between a female (*cot*/*cot*) and a male (*ok*/*ok*) produced F1 offspring (*cot*/*+ ok*/*+*), and then F2 progeny from an intercross of the F1 generation, from which the individuals with genotype (*cot*/*cot ok*/*ok*) and (+/+ *ok*/*ok*) were separated for later use by the cuticle and temperature-treatment method. All larvae were raised on fresh mulberry leaves at 25°C and 60% relative humidity under “long-day conditions” (16 h light: 8 h dark). Eggs were immediately hatched after egg laying.

### Behavioral observations

Dazao (+/+) and *cot* (*cot*/*cot*) silkworms were rubbed with two hands for several seconds to induce the *cot* phenotype at room temperature. To determine whether mechanical or thermal stimulation with hands induced the *cot* phenotype, *cot* mutants were repeatedly rubbed with a cotton bud or placed in an incubator. Larvae of different genotypes (*cot/cot* and +/+) were exposed to 35°C in an electrothermal incubator and maintained for 10 min. Silkworm behavior was recorded by camera (Canon xi810, Japan).

### Heartbeat rhythm

As the *cot* strain (*cot*/*cot ok*/*ok*) has a transparent cuticle, we chose to count the heartbeat rhythm of the dorsal vessel. To confirm that the *cot* strain (*cot*/*cot ok*/*ok*) had the seizure behavior of the *cot* strain (*cot*/*cot*), seizure induction was carried out as previously described. Briefly, hyperthermia-induced seizures were provoked using a temperature-controlled electrothermal incubator. We transferred 15 individuals of each strain (*cot*/*cot ok*/*ok*, +/+ *ok*/*ok* and +/+ +/+) to an incubator at 35°C for 10 min. Seizures were defined as a period of body contract, followed by a failure to maintain standing posture, with rolling, vomiting gut juice and a momentary cessation of movement. The status of individual flies was determined at 1 min intervals and the cumulative number of individuals with seizure was counted at each time point. Meanwhile, the *cot/cot ok/ok* and *+/+ ok/ok* strains were immerged into 42°C for 3 min. Then, the heartbeats were immediately counted. All tests were done with three biological replicates.

### RNA extraction and Illumina sequencing

After hatching, neonatal larvae of each strain were divided into two groups. One group was treated for 5 min at 42°C and the other was placed at room temperature (25°C) and used as a control group. Each sample used RNA pooled from a mixture of 20 neonatal silkworm larvae. Total RNA was extracted using TRIzol Reagent (Invitrogen), purified with absolute alcohol and treated with DNase. An Agilent Bioanalyzer 2100 (Agilent Technologies) was used to assess the integrity/quality of the mRNA. Following the TruSeq RNA Sample Preparation v2 Guide (Illumina), mRNAs were enriched using oligo (dT) magnetic beads and fragmented into short pieces in fragmentation buffer. Then, cDNAs were synthesized using random hexamer primers. Finally, sequencing adaptors were ligated to the cDNA fragments by PCR amplification. Sequencing analysis was performed using a HiSeq 2000 (Illumina) with Beijing Biomarker Technologies CO., LTD (Beijing, China). Raw data presented in this publication have been deposited in the NCBI Short Read Archive (http://www.ncbi.nlm.nih.gov/sra/) and are accessible through SRA accession number: SRP045124.

### Data analysis

As an initial quality control measure, we examined the quality value and base distribution of the raw data. The quality value of a sequence was used to assess the error rate of bases; higher quality values indicated that the corresponding nucleotide base had a lower error rate. Theoretically, the GC and AT content should be equal in each sequencing cycle based on the random interruption of sequence and complementary base pairing principles, and the base distribution curve should be horizontal and stable throughout the sequencing process. The silkworm genome sequence was obtained from SilkDB (http://silkworm.swu.edu.cn/silkdb/). Clean reads were mapped to the silkworm reference sequences using Tophat software [14]. Gene expression levels were calculated using the reads per kb per million reads (RPKM) method [15]. Genes with a False Discovery Rate (FDR) <0.01 and log2 ratios ≥1 were defined as differentially expressed genes (DEGs). DEG sequences were blasted, mapped, annotated and analyzed using the Kyoto Encyclopedia of Genes and Genomes (KEGG) metabolism pathway and the Blast2GO software (version 2.7.2) following the Blast2GO Tutorial [16]. The default settings for Blast2GO were used at every step. Additionally, some genes were annotated using NCBI Blast (http://www.ncbi.nlm.nih.gov/).

### Real-time qRT-PCR

Total RNA from each sample of a mixture of 20 whole neonatal larvae was extracted using TRIzol reagent (Invitrogen) and purified with absolute alcohol. Reverse transcription was made from total RNA (1 µg) with the PrimeScript RT reagent Kit (RR037A, Takara) in a 10 µL reaction volume. The cDNA pool was diluted (1∶10) with distilled water. Gene specific assays were used to quantify genes with the SybrGreen method using Premix Ex Taq II (Takara, RR820A) on CFX96 Real-Time System (Bio-Rad, Hercules, CA, USA) using the default parameters. Samples were run in triplicate and contained 1.5 µL 2 µM specific primers, 7.5 µL SYBR Premix Ex Tag II (Tli RNaseH Plus, Takara; 2×) and 2 µL cDNA; RNase-free water was added to achieve a final volume of 15 µL. The housekeeping gene *Ribosomal protein L3* (*RPL3*) was used as an RNA loading control, as previously described [17]. Data were transformed and analyzed according to the 2^−ΔΔCt^ method [18] and ratios after normalization were expressed as fold-changes compared to control samples. The primers used are listed in Table S1.

## Results and Discussion

### *cot* is a temperature sensitive mutant with a seizure phenotype

In 1996, Fujii *et al.* [13] found that *B. mori cot* larvae had slightly short and stout bodies compared to wild-type larvae. *cot* larvae strongly contracted their bodies when touched, sometimes rolling, vomiting gut juice or a momentary cessation of movement (File S1). Touching by hand produces mechanical and thermal stimuli. However, simple mechanical touch did not induce the *cot* phenotype when *cot/cot* larvae were rubbed with a cotton bud at room temperature. Thus, we induced the mutant phenotype by thermal stimuli. Dazao (+/+) and *cot/cot* larvae were treated with high temperature on day 5 of the fifth instar stage. When *cot/cot* larvae were maintained at 35°C for longer than 3 min, they displayed an epilepsy phenotype that included contraction of the body, rolling and vomiting gut juice. The Dazao (wild-type) larvae showed no *cot/cot* phenotype (File S2). These results indicated that temperature could induce the *cot/cot* epilepsy phenotype.

The *cot* mutant also showed the epilepsy phenotype in the neonatal larvae, pupa and moth stages. When neonatal larvae were thermally stimulated (42°C), *cot* larvae showed strong shaking of the body, turning, loss of motion and vomiting of gut juice (Figure S1). When *cot* larvae were treated with high temperature from the first to the fifth instar stage, they also showed this phenotype and recovered slowly at room temperature. The tail trembled acutely after treatment with high temperature during the pupa stage. Upon reaching adulthood, *cot* moths fell sideways with erect wings and closed legs upon temperature treatment (Figure S1).

### Hyperthermia significantly increases heartbeat in *cot* mutants

**Figure 1:**
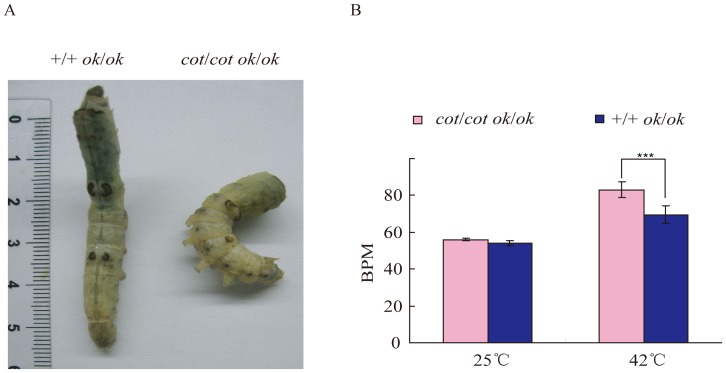
The heartbeat of *cot* mutants. (A) The transparent cuticle phenotype in *cot* mutants (*cot/cot ok/ok*) and controls (*+/+ ok/ok*). (B) The heartbeat of *cot/cot ok/ok* and *+/+ ok/ok* strains at room temperature and after 42°C for 3 min. Ten individuals were investigated for each genotype group with three replicates. BPM, beats per minute. Error bars indicate the SEM. Statistically significant differences were identified using Student's *t*-test; p<0.05.

Seizures can provoke severe cardiac arrhythmias [19]. The *cot* mutant had a seizure phenotype, so we examined heart rhythms after applying a temperature stimulus. The *cot/cot* strain had a white body color that prevented pulse measurements during body contractions. Therefore, we obtained a transparent cuticle *cot/cot* o*k/ok* mutant that was identified from the F2 generation of a F1 *ok/ok*×*cot/cot* intercross (Figure 1). The heartbeat was easily detected in the *cot/cot* o*k/ok* and *+/+ ok/ok* silkworms. The two strains had heartbeats of ∼60 beats per minute (BPM) at room temperature. After 42°C for 3 min, the BPM increased in both strains, but the BPM of the *cot/cot ok/ok* individuals increased more significantly than in the *+/+* o*k/ok* individuals (Figure 1). Thus, *cot* mutants might be helpful for studying the relationship between seizure and cardiac arrhythmias.

### Digital gene expression (DGE) profiles

**Figure 2:**
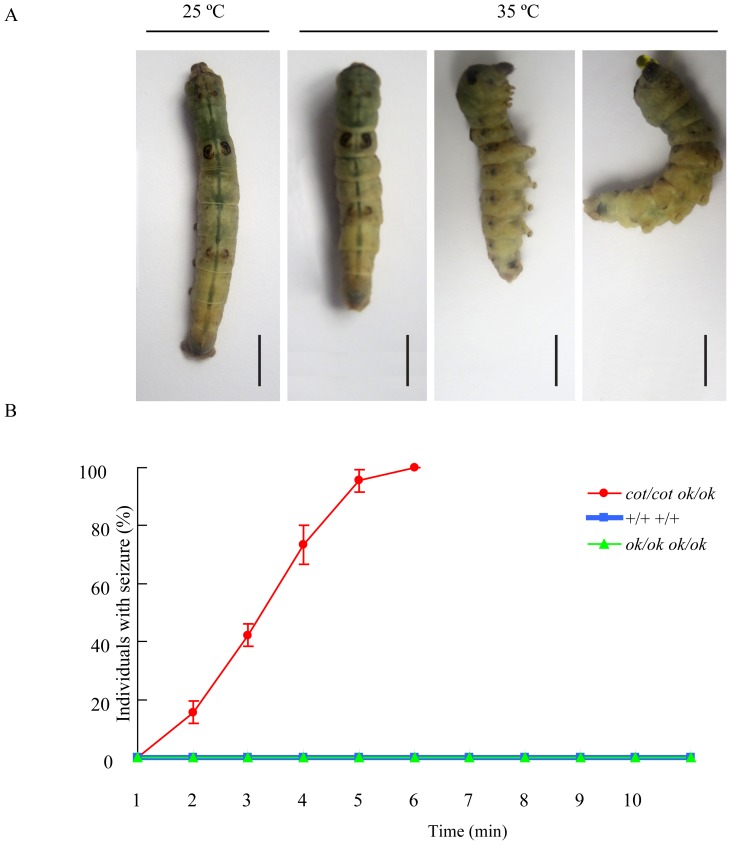
The *cot* mutant (*cot/cot ok/ok*) shows a temperature-induced seizure phenotype. (A) The seizure phenotype of *cot* mutants (*cot/cot ok/ok*) treated at 35°C for 10 min. (B) The behavioral response to hyperthermia in the *cot/cot ok/ok*, *+/+ ok/ok* and *+/+ +/+* strains. Seizures were defined as a period of body contact, followed by failure to maintain standing posture, with rolling, vomiting gut juice and occasional temporary absence of movement. Individuals at day 5 of the fifth instar (n = 15 per strain) were immersed in a temperature-controlled electrothermal incubator (35°C, 10 min). The status of individual flies was determined at 1 min intervals and the cumulative number of individuals with seizure was determined at each time point for each strain. Data are presented as the means ± SEM.

To confirm that the *cot*/*cot ok*/*ok* strain also had a heat-induced seizure phenotype, the *cot*/*cot ok*/*ok*, +/+ *ok*/*ok* and +/+ +/+ strains were observed in a plastic box after immersion in a box in a 35°C electrothermal incubator. The *cot* strain strongly contracted their body after immersion in the 35°C box for 2 min, followed by falling down sideways on account of cramping, along with bending of the body and rolling, vomiting gut juice and sometimes a temporary lack of movement (Figure 2). However, the Dazao (+/++/+) and *ok* (+/+*ok*/*ok*) strains only slightly contracted their bodies at 35°C compared to those at 25°C (Figure S2). All *cot* strains induced by hyperthermia (35°C) had an epilepsy phenotype at the 6 min time point, whereas the other strains did not show it, even after treatment for 10 min (Figure 2). Our results showed that the *cot/cot ok*/*ok* strain had the same seizure phenotype as the *cot*/*cot* strain under hyperthermia.

To investigate transcriptional changes related to the *cot* mutant exposed to hyperthermia, we constructed six DGE tag libraries, numbered correspondingly as *cot* (*cot/cot ok*/*ok*)/25°C, *cot* (*cot/cot ok*/*ok*)/42°C, *ok* (+/+ *ok*/*ok*)/25°C, *ok* (+/+ *ok*/*ok*)/42°C, Dazao (+/+ +/+)/25°C and Dazao (+/+ +/+)/42°C. The Dazao and *ok* strains served as the control groups. Libraries were generated using RNA samples isolated from the hyperthermia-induced (42°C) and control (25°C) silkworm larvae of the three strains and analyzed by high-throughput sequencing with HiSeq 2000 (Illumina). We generated 10.1–10.8 million raw reads for each library (Table 1). The quality score and base distribution showed that Q30 >85% in the six samples and the GC and AT content was almost equal in each sequencing cycle, with a horizontal and stable curve throughout the sequencing process (Table 1 and Figure S3). These results demonstrated that our sequencing quality was robust. After filtering, 89.44%–94.60% clean reads were matched to the silkworm genome using Tophat software [14] and 95.92%–96.48% of the reads in all of the six libraries were uniquely mapped to silkworm sequences (Table 1). To determine whether the sequencing depth was sufficient for transcript coverage or not, we analyzed the sequencing saturation of each library. Our results showed that the number of detected genes mapped by clean reads became saturated when the total read number reached approximately 2.5 M (Figure S4).

**Table 1:** Statistical analysis of transcriptome sequencing data.

Category	Parameter	*cot* (*cot/cot ok/ok*)		*ok* (*+/+ ok/ok*)		Dazao (+/+ +/+)	
		42°C	25°C	42°C	25°C	42°C	25°C
**Raw Data**	Total reads	10,815,099	10,889,282	10,791,854	10,378,568	10,193,219	10,877,363
	Total bases	648,831,340	653,283,371	647,429,398	622,637,928	611,507,003	652,563,266
	GC%	49.06	48.12	48.72	49.66	49.72	48.76
	Q30%	87.41	86.23	85.64	86.44	85.31	86.68
**Clean reads**	Total reads	9,623,567	9,568,099	9,405,424	9,171,645	8,863,802	9,280,269
	Total bases	577,347,396	574,020,574	564,253,527	550,230,453	531,751,922	556,749,076
	GC%	48.53	47.59	48.21	49.16	49.19	48.13
	Q30%	94.91	95.03	94.97	94.95	94.88	94.97
**Mapping to Genome**	Total reads	9,623,567	9,568,099	9,405,424	9,171,645	8,863,802	9,280,269
	Mapped reads	8,658,869 (89.98%)	8,557,478 (89.44%)	8,477,976 (90.14%)	8,313,992 (90.65%)	8,390,896 (94.66%)	8,743,035 (94.21%)
	Unique mapped reads	8,306,440 (95.93%)	8,208,461 (95.92%)	8,111,740 (95.68%)	8,002,545 (96.25%)	8,095,799 (96.48%)	8,418,522 (96.29%)
	Multiple mapped reads	352,429 (4.07%)	349,017 (4.08%)	366,236 (4.32%)	311,447 (3.75%)	295,097 (3.52%)	324,513 (3.71%)

### Analysis of differentially expressed genes

DEGs associated with hyperthermia in the *cot* mutant might provide new insights into the molecular mechanism of seizure induced by hyperthermia. Based on RPKM methods, the expression levels of 14,623 annotated silkworm genes were determined in each of the six samples (Table S2). We used an FDR <0.01 and a log2 ratio ≥1 as a threshold to identify DEGs. The number of DEGs in each strain induced with hyperthermia (42°C) for 5 min was different compared to those maintained at 25°C. The number of DEGs was 147, 221 and 24 in the *cot* (*cot/cot ok*/*ok*), *ok* (+/+ *ok*/*ok*) and Dazao (+/+ +/+) strains, respectively (Table S3). There were only 24 DEGs in the Dazao strain, suggesting that it is a heat-resistant strain.

**Figure 3:**
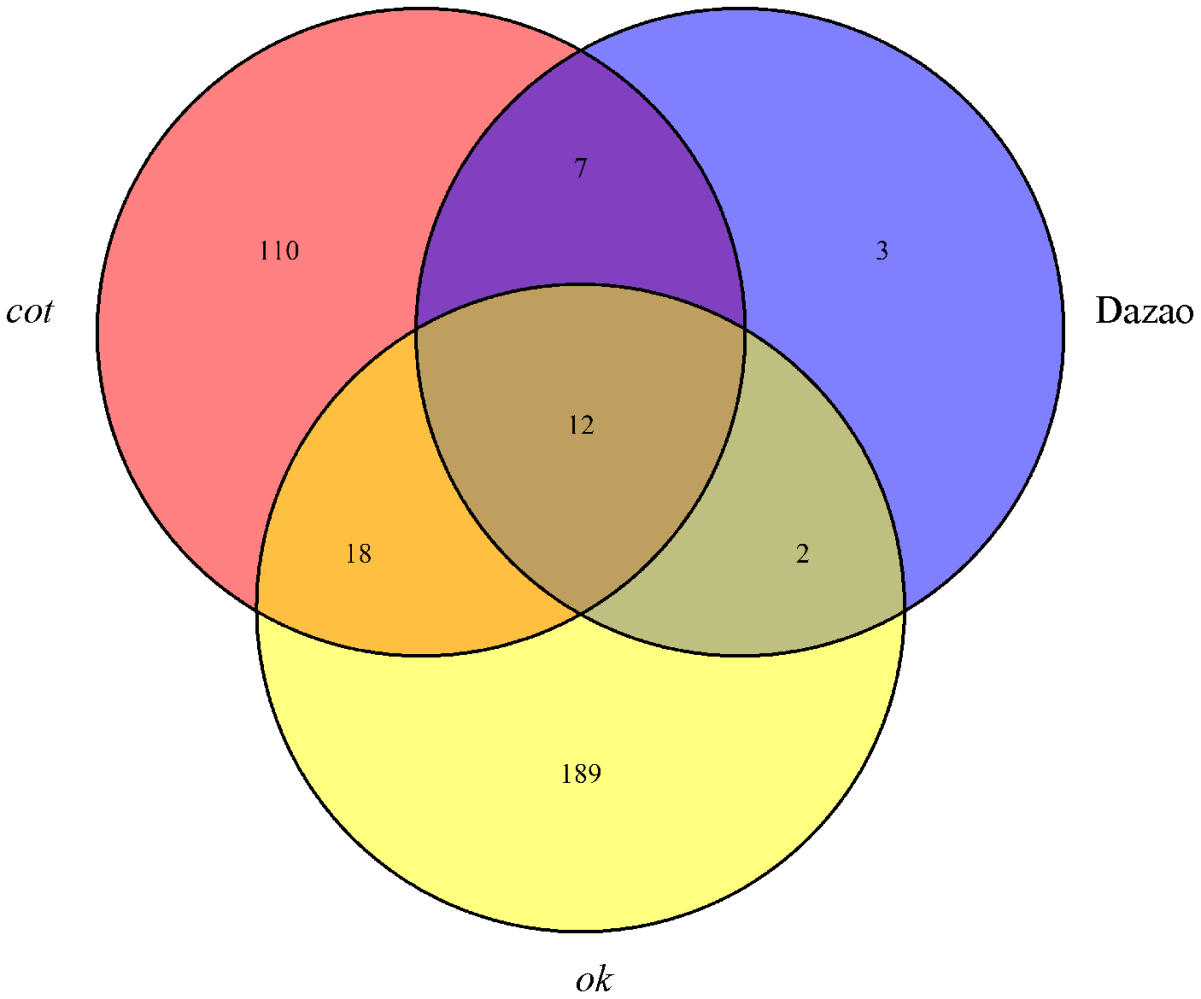
The number of common DEGs between different strains after hyperthermia-induced treatment. To quantify the overlap in the response to high temperature, a Venn diagram was constructed. The DEGs of the *cot* (*cot/cot ok/ok*) strain treated with hyperthermia compared to individuals at 25°C are indicated by “*cot.*” The DEGs of the *ok* (*+/+ok/ok*) strain treated with hyperthermia compared to individuals at 25°C are indicated by “*ok.*” The DEGs of the Dazao (+/+ +/+) strain treated with hyperthermia compared to individuals at 25°C are indicated by “Dazao.”

A Venn diagram analysis of the DEGs among the different strains revealed that 12 common genes showed differential expression patterns in all of the three strains exposed to hyperthermia (42°C) for 5 min; 30 genes showed differential expression in both the *cot* (*cot/cot ok*/*ok*) and *ok* (+/+ *ok*/*ok*) strains, and 19 genes showed differential expression in both the *cot* (*cot/cot ok*/*ok*) and Dazao (+/++/+) strains (Figure 3). Among the 12 most common genes, 11 were up-regulated, and only one (*BGIBMGA004290*) transcript that encodes an annotated uncharacterized protein (LOC100216500) precursor was down-regulated (Table 2). Furthermore, 9 up-regulated genes were annotated HSPs and the other 2 genes were lethal proteins that are essential for life, [*l(2)efl*] and *DnaJ-5*. The gene *l(2)efl* belongs to the small HSP family [20] and *DnaJ*, also known as HSP40, is a crucial partners for Hsp70 chaperones [21]. HSPs play a critical role in the development of thermotolerance and protection from cellular damage associated with stresses, such as hyperthermia [22]. Most HSPs were significantly up-regulated in the 3 strains after treatment with hyperthermia, suggesting that they have an important role in response to high temperature in silkworms.

**Table 2:** Common differentially expressed genes in 3 strains (*cot/cot ok*/*ok*, +/+ *ok*/*ok* and +/+ +/+) after treatment with hyperthermia (42°C).

Gene ID	NCBI Blast_annotation	Regulated	log_2_Fold^a^	log_2_Fold^b^	log_2_Fold^c^
BGIBMGA001635	PREDICTED: heat shock protein 68-like [*Bombyx mori*]	up	5.79	10.79	5.20
BGIBMGA004515	heat shock protein hsp23.7 precursor [*Bombyx mori*]	up	3.10	8.64	3.39
BGIBMGA004540	heat shock protein hsp 19.9 [*Bombyx mori*]	up	4.78	9.18	4.72
BGIBMGA004541	heat shock protein 20.4 [*Bombyx mori*]	up	4.77	10.39	5.49
BGIBMGA004605	heat shock protein hsp20.8 [*Bombyx mori*]	up	4.30	9.20	5.00
BGIBMGA004613	PREDICTED: heat shock protein 68-like [*Bombyx mori*]	up	5.47	9.83	7.24
BGIBMGA004614	PREDICTED: heat shock protein 70 A2-like [*Bombyx mori*]	up	7.15	10.47	7.23
BGIBMGA005780	PREDICTED: protein lethal(2)essential for life-like [*Bombyx mori*]	up	4.68	10.83	5.46
BGIBMGA013536	DnaJ-5 [*Bombyx mori*]	up	1.13	3.86	1.45
BGIBMGA014536	heat shock protein 68 [*Bombyx mori*]	up	7.96	10.90	7.41
BGIBMGA014618	heat shock protein 70 [*Bombyx mori*]	up	6.82	10.87	7.64
BGIBMGA004290	uncharacterized protein LOC100216500 precursor [*Bombyx mori*]	down	−1.86	−2.04	−1.28

Note: Fold^a^ indicates the ratio of RPKM of *cot*/*cot ok*/*ok* (42°C) sample divided by the RPKM of *cot*/*cot ok*/*ok* (25°C) samples; Fold^b^ indicates the ratio of RPKM of *+/+ ok/ok* (42°C) sample divided by the RPKM of *+/+ ok/ok* (25°C) samples; Fold^c^ indicates the ratio of RPKM of *+/+ +/+* (42°C) sample divided by the RPKM of *+/+ +/+* (25°C) samples.

**Figure 4:**
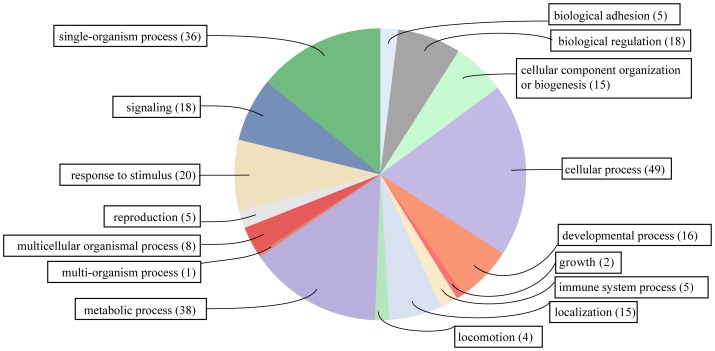
The category of biological process for the unique DEGs in the *cot* strain after hyperthermia induction. DEGs (Table S4) were broadly categorized according to their biological process. Numbers represent the actual number of DEGs.

To identify DEGs related with hyperthermia in the *cot* strain, 110 DEGs that were exclusively detected in the *cot* strain were selected for subsequent analysis (Table S4). Among these 110 DEGs, 108 were up-regulated and two were down-regulated in the hyperthermia (42°C)-induced *cot* sample compared to the untreated *cot* samples; these DEGs were subjected to functional annotation using Blast2GO. Gene ontology (GO) assignments were used to analyze the functions of the DEGs. The DEGs were classified into 16 biological process categories (Figure 4) using Blast2GO, including genes related to cellular processes (49 genes), metabolic processes (38 genes), single-organism processes (36 genes), responses to stimuli (20 genes), signaling (18 genes) and biological regulation (18 genes).

To identify the biological pathways that are active in *cot* mutant induced-hyperthermia, the DEGs were mapped to the reference canonical pathways in the KEGG database [23]. Among the 110 unique DEGs in the *cot* mutant, 27 sequences predicted to encode enzymes with enzyme commission (EC) numbers were mapped to 15 different KEGG pathways (Table S4), which are illustrated in Table S5. The top three KEGG pathways among the DEGs were “Biotin metabolism,” “Fatty acid biosynthesis” and “Purine metabolism,” implying that these diverse metabolic processes are active in *cot* mutant induced-hyperthermia.

### Validation of selected genes by qRT-PCR

To validate the transcriptome results, 12 common DEGs and 10 unique DEGs were selected for qRT-PCR verification (Table S6). Generally, the trends for down- or up-regulated transcription of the DEGs by qRT-PCR analysis were consistent with those of the DGE expression profiling analysis, indicating the reliability of our DGE expression profiling analysis.

### Differentially expressed genes in the ubiquitin system

Epilepsy is associated with the insertion, deletion and substitution of mutant genes [24]–[26], which generate mutant proteins. The degradation of mutant proteins is critical for the regulation of many diverse cellular processes. The majority of protein degradation in cells occurs via the ubiquitin-proteasome system [27]. The *cot* mutation is a behavioral mutant, which has a seizure phenotype after hyperthermia. Among the differentially expressed genes, *BGIBMGA002161*, *BGIBMGA005660* and *BGIBMGA009345* (encoding Cullin-2, Low quality protein: putative E3 ubiquitin-protein ligase UNKL-like and E3 ubiquitin-protein ligase SH3RF1-like, respectively) are involved in the ubiquitin-proteasome system and were up-regulated (Table 3). The ubiquitin protein is attached to a substrate through the action of three enzymes: ubiquitin-activating enzyme (E1), ubiquitin-conjugating enzyme (E2) and ubiquitin ligases (E3s). Among these enzymes, ubiquitin ligases (E3) provide substrate specificity for ubiquitination. The largest known class of ubiquitin ligases are the cullin-RING ubiquitin ligases [28], which are multi-subunit complexes that contain a cullin, a RING H2 finger protein, a substrate-recognition subunit and an adaptor subunit [29]. *Cullin-2*, which is an ubiquitin ligase subunit, was up-regulated in *cot* mutants after hyperthermia, suggesting that ubiquitin ligases may be up-regulated to degrade the product of the mutant gene. Furthermore, loss-of-function of Cullin-2 increased the number of boutons on a single axon at larval neuromuscular junctions and neurons [30]. Boutons contact with the cell body and dendrites of other neurons, forming a synapse. In our study, the up-regulation of *cullin-2* may reduce the number of boutons, causing abnormal signal transduction in *cot* mutants induced by hyperthermia. C-terminus of Hsp70-interacting protein, an E3 ubiquitin ligase, can also be up-regulated to degrade mutant proteins [31]. Two ubiquitin ligase genes (*BGIBMGA005660* and *BGIBMGA009345*) were up-regulated in the *cot* mutant, suggesting that ubiquitin ligases may play an important role in protein degradation under hyperthermia conditions. Additionally, *BGIBMGA005053*, which encodes a deubiquitinating enzyme (ubiquitin carboxyl-terminal hydrolase 34), was up-regulated in the *cot* mutant (Table 3). Deubiquitinating enzymes have four important functions in the ubiquitin pathway: (1) processing of ubiquitin precursors; (2) editing or rescue of ubiquitin conjugates; (3) recycling of ubiquitin or ubiquitin oligomers from ubiquitin–protein conjugates targeted for degradation; and (4) disassembly of unanchored ubiquitin oligomers [32]. Therefore, the up-regulated gene *BGIBMGA00505* encodes an enzyme that not only can remove ubiquitin from proteins inappropriately targeted to the proteasome, but can also process inactive ubiquitin precursors to maintain a sufficient pool of free ubiquitin in the ubiquitin system.

**Table 3:** Unique DEGs of interest in the *cot* mutant (*cot/cot ok*/*ok*) treated with hyperthermia.

Classification	Gene ID	log_2_Fold^a^	Regulated	NCBI Blast/nr_annotation
**The ubiquitin system**	BGIBMGA002161	1.27	up	cullin-2
	BGIBMGA005660	1.81	up	Low quality protein: putative E3 ubiquitin-protein ligase UNKL-like [*Bombyx mori*]
	BGIBMGA009345	1.54	up	E3 ubiquitin-protein ligase SH3RF1-like [*Bombyx mori*]
	BGIBMGA005053	2.17	up	ubiquitin carboxyl-terminal hydrolase 34
**Nicotinic acetylcholine receptors**	BGIBMGA004521	1.97	up	nicotinic acetylcholine beta-3 subunit [*Bombyx mori*]
	BGIBMGA007809	1.46	up	nicotinic acetylcholine receptor subunit alpha 9
**Cardiac excitation–contraction coupling**	BGIBMGA008283	1.28	up	phospholipase C-beta
	BGIBMGA013346	2.30	up	voltage-gated calcium channel [*Loa loa*]
	BGIBMGA013392	1.17	up	inositol 1,4,5-trisphosphate receptor-like [*Bombyx mori*]
	BGIBMGA013297	2.27	up	ryanodine receptor
	BGIBMGA004531	1.36	up	plasma membrane calcium-transporting ATPase 3
	BGIBMGA002239	1.52	up	probable sodium/potassium/calcium exchanger CG1090-like [*Bombyx mori*]
	BGIBMGA009120	1.95	up	Low quality protein: protein 4.1 homolog [*Bombyx mori*]
**Notch signaling pathway**	BGIBMGA007929	1.46	up	Notch homolog [*Bombyx mori*]
	BGIBMGA000852	1.33	up	Low quality protein: histone acetyltransferase p300-like [*Bombyx mori*]

Note: Fold^a^ indicates the ratio of RPKM of *cot*/*cot ok*/*ok* (42°C) samples divided by the RPKM of *cot*/*cot ok*/*ok* (25°C) samples.

### Differentially expressed genes that encode nicotinic acetylcholine receptors

The nicotinic acetylcholine receptor (nAChR) mediates fast synaptic cholinergic transmission in the insect central nervous system [33] and is a pentameric membrane protein that is composed of five subunits organized around a central pore [34]. *B. mori* possesses the largest known insect nAChR gene family, including nine α-type and three β-type subunits [35]. We found that the nicotinic acetylcholine beta-3 subunit and nicotinic acetylcholine receptor subunit alpha-9 (*BGIBMGA004521* and *BGIBMGA007809*) were up-regulated in *cot* mutants induced with hyperthermia (Table 3). In humans, autosomal dominant nocturnal frontal lobe epilepsy was mainly associated with mutations in genes coding nAChR subunits (*CHRNA4*, *CHRNB2* and *CHRNA2*) [36]–[38]. Different nAChR subunits can form a variety of heterologous/homologous pentamers during different developmental stages, resulting in different pharmacological properties [39]. Two genes (nicotinic acetylcholine beta-3 subunit and nicotinic acetylcholine receptor subunit alpha-9) were up-regulated in *cot* mutants, which might form pentamers with different subunits and cause abnormal synaptic transmission compared to other strains.

### Differentially expressed genes in cardiac excitation–contraction coupling

Cardiac excitation–contraction coupling is the process of electrical excitation from the myocyte to the contraction of the heart [40]. The ubiquitous second messenger Ca^2+^ is essential for cardiac electrical activity and is a direct activator of the myofilaments, which cause contraction [40]. Myocyte mishandling of Ca^2+^ is a central cause of both contractile dysfunction and arrhythmias in pathophysiological conditions [41]. Here, six related genes, *BGIBMGA008283*, *BGIBMGA013346*, *BGIBMGA013392*, *BGIBMGA013297*, *BGIBMGA004531* and *BGIBMGA002239* (annotated as phospholipase C-beta, voltage-gated calcium channel, inositol 1,4,5-trisphosphate receptor-like, ryanodine receptor, plasma membrane calcium-transporting ATPase 3 and probable sodium potassium calcium exchanger cg1090-like, respectively), were up-regulated in *cot* mutants (Table 3). The intracellular Ca^2+^ storage/release organelle is the endoplasmic reticulum (ER) in most cells, and is the sarcoplasmic reticulum (SR) in striated muscle cells. The ER and SR contain specialized Ca^2+^ release channels: the ryanodine receptors (RyRs) and inositol trisphosphate receptors (IP_3_Rs) [42]. Mutation of the ryanodine receptor isoform 2, encoding the cardiac Ca^2+^ release channel, causes abnormalities of intracellular Ca^2+^ regulation [43]. Ca^2+^ release by RyRs was triggered though Ca^2+^-induced Ca^2+^-release [40]. Phospholipase C hydrolyzes the membrane lipid phosphatidylinositol 4,5-bisphosphate, generating inositol 1,4,5-trisphosphate (InsP_3_) [44]. InsP_3_ can bind to its receptor (inositol 1,4,5-trisphosphate receptor, InsP_3_R), which is an intracellular ligand-gated Ca^2+^ release channel [45] that is localized primarily in the ER membrane [46]. Upon binding InsP_3_, the InsP_3_R is gated open, resulting in the release of Ca^2+^ stored in the ER lumen into the cytoplasm. The up-regulation of the inositol triphosphate receptor is associated with sensitization to Ca^2+^ release and vascular smooth muscle contractility [47]. Based on the mechanism of cardiac excitation–contraction coupling and related DEGs, we hypothesize a possible explanation for the abnormal contraction of body segments, and the increased heartbeat under high-temperature regimes (>35°C). At temperatures greater than 35°C, Ca^2+^ enters the cell through depolarization-activated Ca^2+^ channels (*BGIBMGA013346*, encoding a voltage-dependent calcium channel) during the action potential. This process triggers Ca^2+^ release from RyRs (*BGIBMGA013297*) and IP_3_Rs (*BGIBMGA013392*); meanwhile, the up-regulation of phospholipase C-beta (*BGIBMGA008283*) might generate more InsP_3_, which could also trigger Ca^2+^ release through InsP_3_ receptors. These two factors raise the free intracellular Ca^2+^ concentration [Ca^2+^]_i_, which then turns on the contraction machinery and causes abnormal contraction of body segments and the dorsal vessel. For relaxation, [Ca^2+^]_i_ must decline and Ca^2+^ must be transported out of the cytosol via the probable sodium potassium calcium exchanger cg1090-like (*BGIBMGA002239*) and plasma membrane calcium-transporting ATPase 3 (*BGIBMGA004531*). Additionally, *BGIBMGA009120* (encoding low quality protein: protein 4.1 homolog) was also up-regulated (Table 3), and 4.1N protein is a brain-specific member of the 4.1 protein family of cytoskeletal proteins [48]. The gene encoding 4.1R was highly expressed in the brain and mutations in 4.1R protein resulted in a neurobehavioral phenotype, suggesting that the 4.1R protein has an important function in the brain [49]. InsP_3_R can specifically bind to 4.1N protein by yeast two-hybrid assays and forms the protein 4.1N–InsP3R complex that is enriched in synapses [50]. These findings suggested that *BGIBMGA009120*, which was up-regulated in *cot* mutants treated with hyperthermia, may play a role in Ca^2+^ signaling in neurons.

### Differentially expressed genes in the Notch signaling pathway

The Notch signaling pathway, which is a phylogenetically conserved signaling pathway, is involved in the regulation of diverse biological processes, including mammalian cardiogenesis, and mutations in Notch signaling are associated with congenital heart disease [51]. As the heartbeat was significantly increased in *cot* mutants treated with hyperthermia, homologous genes involved in Notch signaling pathway genes in silkworms were examined in the unique DEGs in *cot* mutants. We found that *BGIBMGA007929* (a Notch homolog) and *BGIBMGA000852* (histone acetyltransferase p300-like) were up-regulated in *cot* mutants treated with hyperthermia (Table 3 and Figure S5). Upon ligand binding, Notch is activated and releases Notch intracellular domain (NICD) from the cell membrane following two successive cleavage events. When Notch signaling is stimulated, NICD, CSL (C promoter-binding factor L in humans) and Mastermind-Like 1 (MAML) form a tripartate complex, which stabilizes the binding of CSL/NICD to DNA; thereafter, histone acetyltransferase p300 (HATs) can up-regulate target gene transcription [51]. In this study, Notch homolog and histone acetyltransferase p300-like were up-regulated, suggesting that Notch signaling was stimulated and some heartbeat target genes might be up-regulated in *cot* mutants treated with hyperthermia.

Additionally, the overexpression of a dominant-negative Notch or Notch knock-down leads to decreased dendritic branching in cortical neurons [52]. As dendrites are key integrators of synaptic information in neurons, the structure and arborization of dendrites has a vital impact on the processing of neuronal information [53]. We found that the Notch homolog (*BGIBMGA007929*) was up-regulated in *cot* mutants treated with hyperthermia, suggesting that it may have an important role in the processing of neuronal information.

## Supporting Information

Figure S1 **The phenotype of** ***cot*** **neonatal larvae and adults treated with hyperthermia.** (A) Neonatal larvae with the *cot*/*cot* genotype (right) showed strong contractions, rolling and the temporary absence of movement after treatment at 42°C; wild-type (+/+, left) larvae did not show abnormal behavior. (B) Wings of *cot*/*cot* moths (right) were erect at 42°C, while wild-type wings (left) were horizontal.
(TIF)

Figure S2 **The phenotype of the** ***ok*** **(+/+** ***ok*** **/** ***ok*** **) and Dazao (+/+ +/+) strains treated at 35°C for 10 min.**
(TIF)

Figure S3 **Base distribution of the six DGE tag libraries.** Horizontal axis, the base position of reads; vertical axis, the proportion per base normalized to total number of bases at that position.
(TIF)

Figure S4 **Saturation curve of the six samples. Horizontal axis, total tag number (M); vertical axis, total gene number (K). RPKM >0.1 was set a threshold for gene expression.**
(TIF)

Figure S5 **The Notch signaling pathway.** Up-regulated genes in the Notch signaling pathway are indicated by red boxes.
(TIF)

Table S1 **Primer pairs used for qRT-PCR.**
(XLS)

Table S2 **The RPKM of 14,623 annotated silkworm genes in six neonatal larvae samples.**
(XLS)

Table S3 **Number of differentially expressed genes in the three strains (** ***cot/cot ok*** **/** ***ok*** **, +/+** ***ok*** **/** ***ok*** **and +/+ +/+) after 42°C for 5 min.**
(DOC)

Table S4 **The unique DEGs in** ***cot*** **mutants after hyperthermia.**
(XLS)

Table S5 **KEGG pathways of the unique DEGs in** ***cot*** **mutants (** ***cot*** **/** ***cot ok*** **/** ***ok*** **) treated with hyperthermia.**
(XLS)

Table S6 **Verification of DEGs by qRT-PCR.**
(XLS)

File S1 **Rubbing the silkworm by hand.** The silkworms marked by red circles represent the *cot* mutant strain (*cot*/*cot*), and the ones marked by blue circles represent wild-type (+/+) individuals.
(MP4)

File S2 **The larvae were heated at 35°C for 10 min.** The silkworm marked by a red circle represents the *cot* mutant strain (*cot*/*cot*), and the ones marked by blue circles represent wild-type (+/+) individuals.
(MP4)
