# Supplementary figures and images for: Transcriptome Analysis of Neonatal Larvae after Hyperthermia-Induced Seizures in the Contractile Silkworm, Bombyx mori
Source: PLoS One. 2014 Nov 25;9(11):e113214. doi: 10.1371/journal.pone.0113214 (PMC4244138; doi:10.1371/journal.pone.0113214)

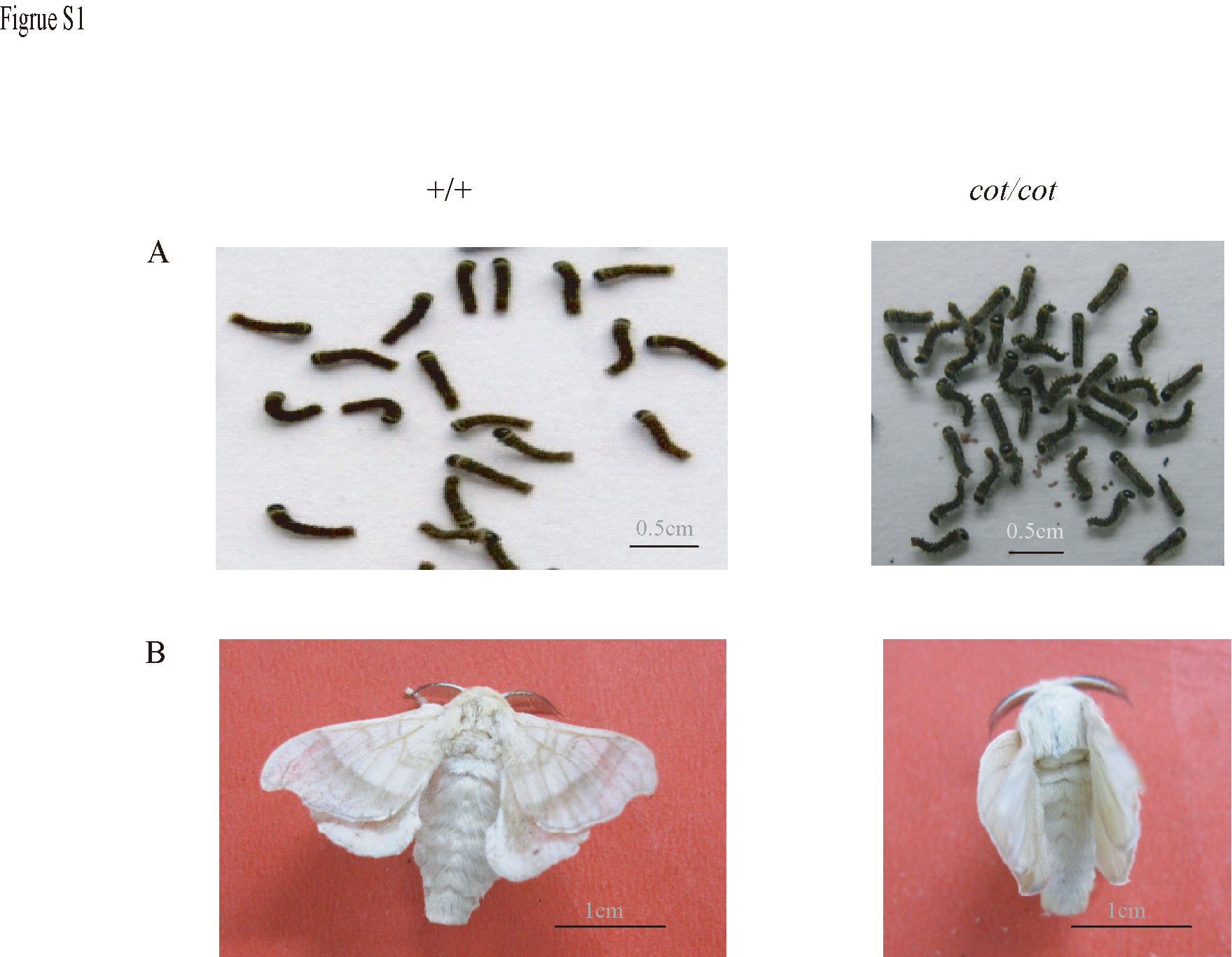

Supplement: Figure S1 — The phenotype of cot neonatal larvae and adults treated with hyperthermia. (A) Neonatal larvae with the cot/cot genotype (right) showed strong contractions, rolling and the temporary absence of movement after treatment at 42°C; wild-type (+/+, left) larvae did not show abnormal behavior. (B) Wings of cot/cot moths (right) were erect at 42°C, while wild-type wings (left) were horizontal. (TIF) [file pone.0113214.s001.tif]

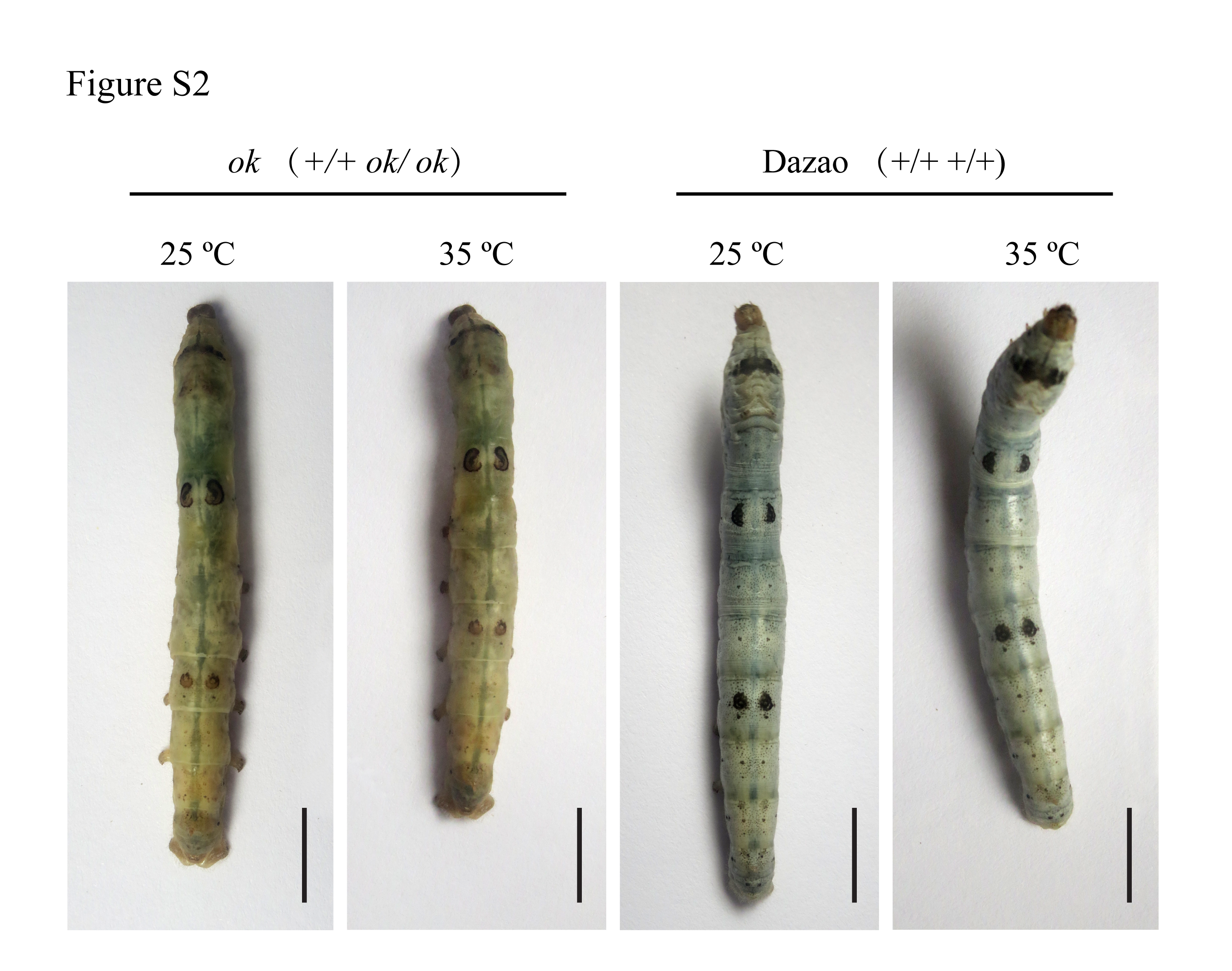

Supplement: Figure S2 — The phenotype of the ok (+/+ ok / ok ) and Dazao (+/+ +/+) strains treated at 35°C for 10 min. (TIF) [file pone.0113214.s002.tif]

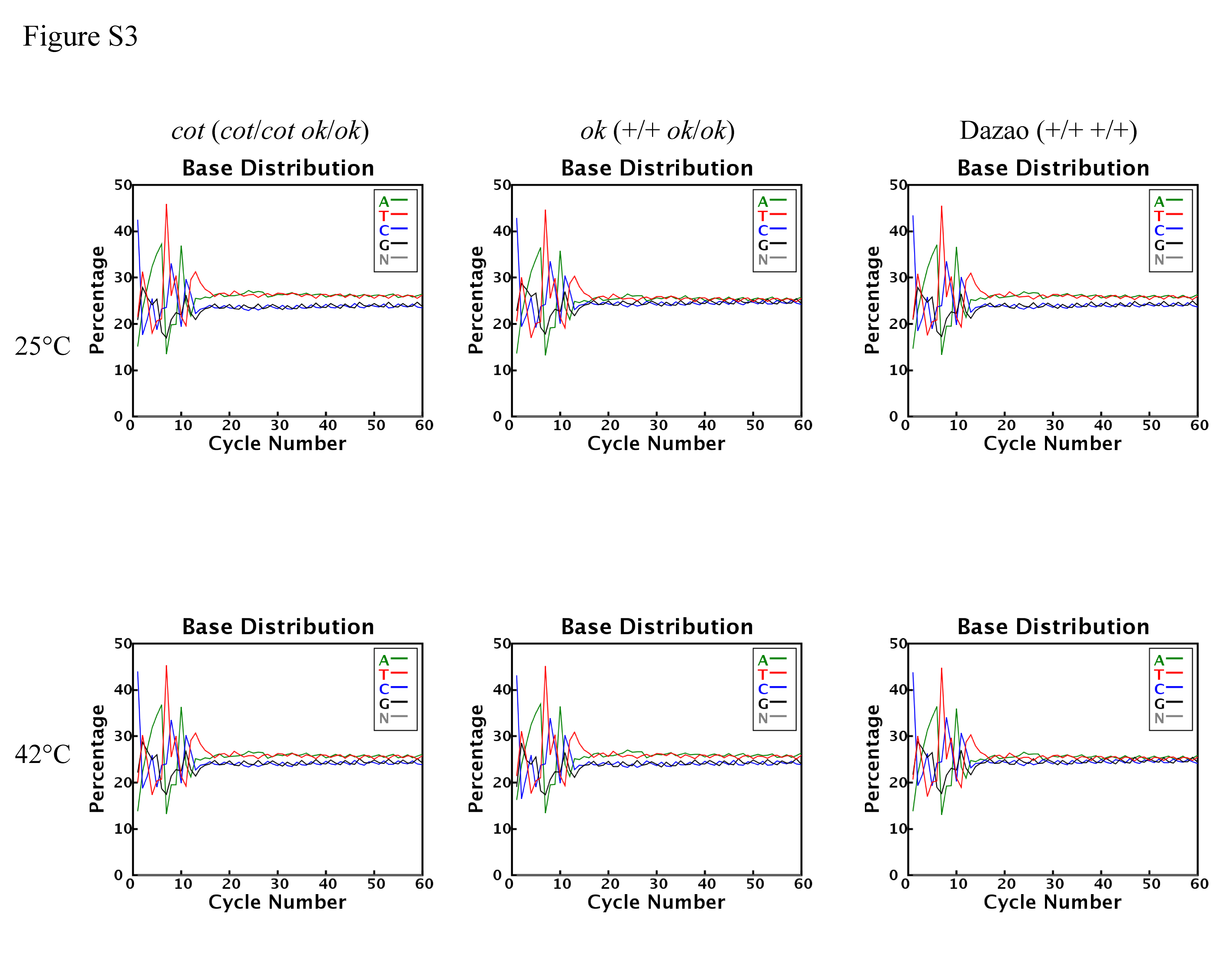

Supplement: Figure S3 — Base distribution of the six DGE tag libraries. Horizontal axis, the base position of reads; vertical axis, the proportion per base normalized to total number of bases at that position. (TIF) [file pone.0113214.s003.tif]

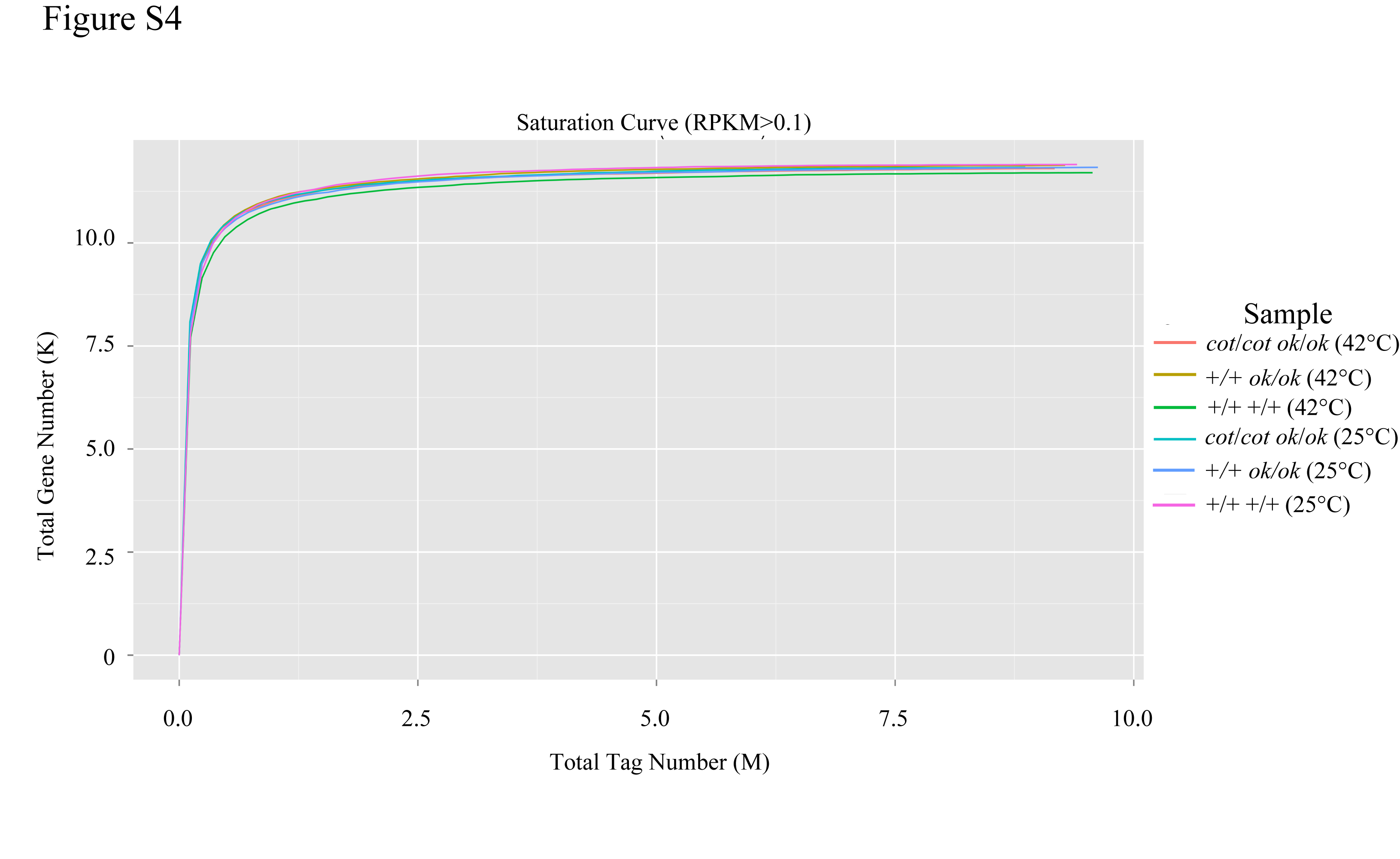

Supplement: Figure S4 — Saturation curve of the six samples. Horizontal axis, total tag number (M); vertical axis, total gene number (K). RPKM >0.1 was set a threshold for gene expression. (TIF) [file pone.0113214.s004.tif]

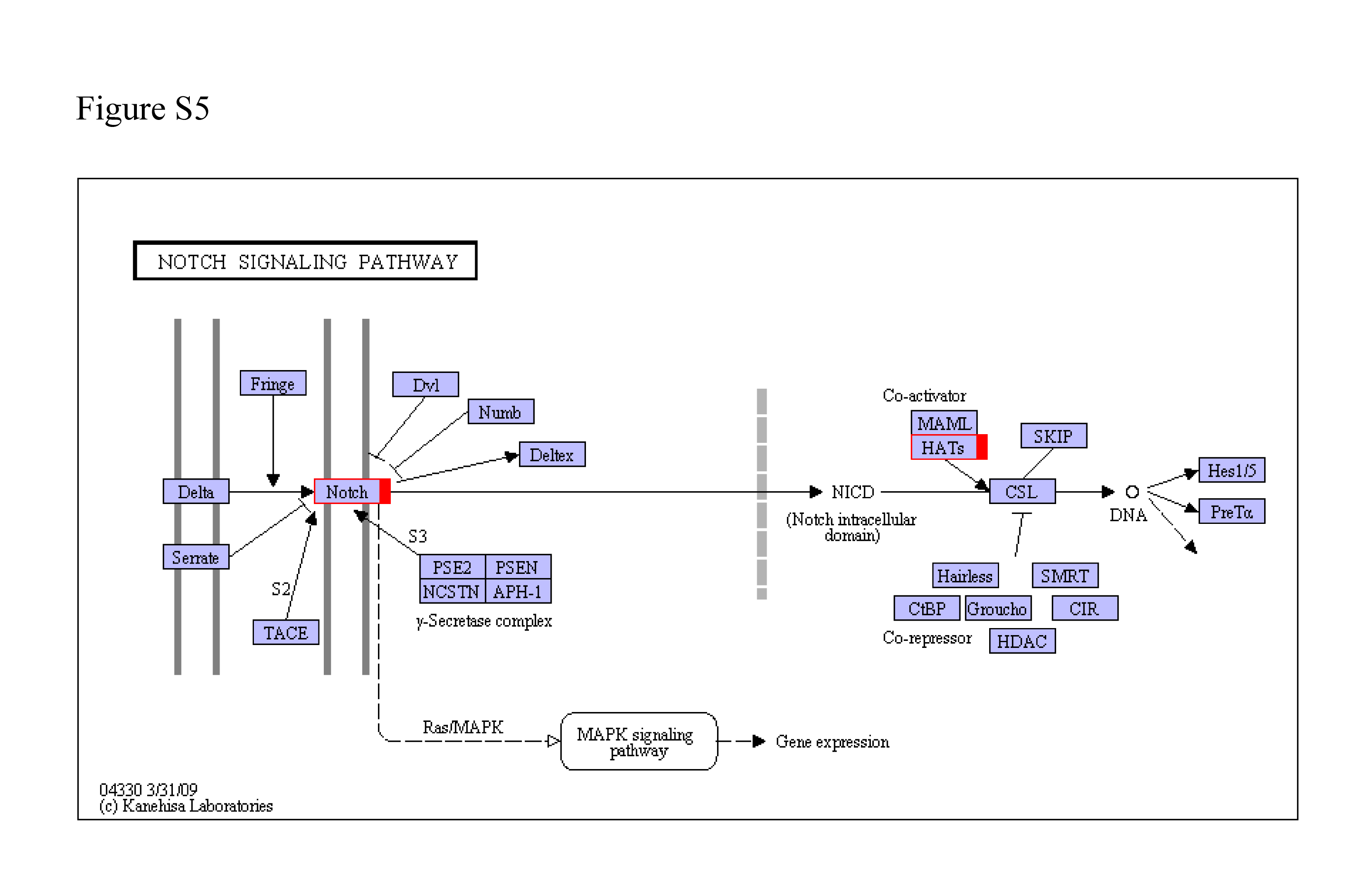

Supplement: Figure S5 — The Notch signaling pathway. Up-regulated genes in the Notch signaling pathway are indicated by red boxes. (TIF) [file pone.0113214.s005.tif]
