# Supplementary material for: Transcriptome Analysis of Neonatal Larvae after Hyperthermia-Induced Seizures in the Contractile Silkworm, Bombyx mori
Source: PLoS One. 2014 Nov 25;9(11):e113214. doi: 10.1371/journal.pone.0113214 (PMC4244138; doi:10.1371/journal.pone.0113214)
Supplement: Table S3 — Number of differentially expressed genes in the three strains ( cot/cot ok / ok , +/+ ok / ok and +/+ +/+) after 42°C for 5 min. (DOC) [file pone.0113214.s008.doc]

**Table S3 Number of differential expressed genes (DEGs) in three strains (*cot/cot* *ok*/*ok*, +/+ *ok*/*ok* and +/+ +/+) after 42°C for 5 min.**

| **Type** | **Total number of DEG** | **The number of DEG** | |
| --- | --- | --- | --- |
|  |  | **up** | **down** |
| *cot* 42°C vs *cot* 25°C | 147 | 144 | 3 |
| *ok* 42°Cvs *ok* 25°C | 221 | 107 | 114 |
| Dazao 42°Cvs Dazao 25°C | 24 | 23 | 1 |
